# P42 Primary Sjogren’s Syndrome (pSS) in a seronegative adolescent female with family history of BehÇet’s disease: A challenging disease to diagnose Case-based discussion

**DOI:** 10.1093/rap/rkac067.042

**Published:** 2022-09-28

**Authors:** Aseel Gharaibeh, Eslam Al-abadi

**Affiliations:** Birmingham Children's Hospital, Birmingham, United Kingdom; Birmingham Children's Hospital, Birmingham, United Kingdom

## Abstract

**Introduction/Background:**

Sjogren’s Syndrome (SS) is heterogenous chronic autoimmune disease with symptoms ranging from organ-specific to variable non-specific systemic features. It is a rare disease in childhood and can be very difficult to diagnose, especially in seronegative patients, with the absence of any specific diagnostic investigation or criteria. We are presenting a seronegative juvenile Primary Sjogren’s syndrome (pSS) patient with all the challenges we had to confirm the diagnosis and a highlight on the role of MDT as the most sensitive tool.

**Description/Method:**

A 14-year-old female patient was first referred to our Paediatric and adolescent rheumatology department at the age of 10 with history of recurrent bilateral painful parotid swellings since the age of 3 with absence of ANA, RF, Anti-SSA-Ro and Anti-SSB-La antibodies repeatedly. From 2016 till 2019 she was under the care of ENT with recurrent complicated bilateral ear infections. Episodes tend to occur every 2-3 weeks and last between a few days to 2 weeks. Her events were associated with fever, fatigue, shivering, joint and leg pain, cervical lymphadenopathy, infrequent folliculitis, and headaches. No history to indicate mouth or eye dryness or significant repeated ulcers. Both of our patient’s mother and maternal grandmother have Behçet’s disease. Further blood investigation only showed mild hypergammaglobulinemia with mildly elevated ESR and CRP, normal LFT, U&Es, complements, muscle enzymes and thyroid function. HIV serology was negative and serum IgG4 subclass was normal. Many USSs confirmed parotitis. Parotid gland biopsy was not specific and indicated chronic parotitis with diffuse infiltration of mature lymphocytes, periductal lymphocytic inflammation and exocytosis of lymphocytes into the ductal epithelium. Focus score nearly reaching 50 cells. Plasma cell staining ruled out IgG4 related disease. Ophthalmic assessment ruled out eye involvement by the appearance of her eyes. The Oral Medicine Team confirmed the appearance of dry mucosa with minimal salivary flow from the parotid ducts, though it has not been subjectively reported to cause any problem before. Failed trial of colchicine, never been off-prednisolone for at least the last 2 years, tried Methotrexate which resulted in significant but not full improvement. Now she is on azathioprine and Hydroxychloquine, but still reporting flares. Escalation in treatment is required though there are some concerns about previous medication compliance, the actual severity of symptoms reported and the lack of current suitable accommodation.

**Discussion/Results:**

Diagnosing pSS is quite challenging especially in seronegative patients due to the possibility of other non-autoimmune diseases causing recurrent parotid gland swellings, especially Recurrent Juvenile Idiopathic Parotitis with very young age of onset. With the persistence of symptoms and lack of an underlying infection, our patient ended up having a parotid biopsy. This was generally not specific, with a focus score close to the 50 mononuclear cells required as evidence of exocrine inflammation. This further added to the challenge of confirming an underlying autoimmune disease. Nine years into the disease course, our patient was confirmed to have objective evidence of dry mucosa and decreased salivary flow from the parotid ducts. This shows how important it is to assess salivary and lacrimal gland dysfunction in a child with suspected pSS, despite the absence of typical xerophthalmia and xerostomia. Considering the non-typical features (sero-negativity and the non-specific histopathology findings), our patient clinical picture was discussed in an MDT meeting including ENT, Histopathology and Rheumatology and the diagnoses of pSS was confirmed. The chronicity of the disease, significant improvement of the flare frequency and severity while on immunosuppressants, the reoccurence of significant flares when the patient was off-treatment (personal decision), and the objective evidence of poor salivary flow all supported the diagnosis of pSS later in the disease course. This case shows us how the typical adult features of pSS should not be strictly applied to the juvenile form of the disease. It also highlights the importance of having an MDT as the most sensitive available tool to confirm the diagnosis.

Regarding lack of subjective full response, how do you think we can prove this objectively and what would be the most appropriate next step in treatment escalation?

**Key learning points/Conclusion:**

A high index of suspicion is crucial to reach the diagnosis and to avoid irreversible damage in children with SS.

Quadrable sero-negativity should not be looked at as an absolute exclusion criterion of juvenile pSS.

Exclusion of other more common and diverse causes of parotitis is crucial.

The typical adult features of SS should not be strictly applied to the juvenile form of the disease.

MDT approach is the most sensitive tool to diagnose SS.

When there is no subjectively reported ophthalmic or oral dryness, we would advocate sending suspected children of pSS for formal assessment of it- we understand the age-related challenges.

Considering its rarity, we need to improve the general awareness of the juvenile form of the disease among the population and the medical practitioners.

